# Case Report: Rapid progression following fertility-sparing management of high-grade endometrial carcinoma

**DOI:** 10.3389/fmed.2025.1619601

**Published:** 2025-07-30

**Authors:** Wang Qilin, Liu Yang, Li Junqiang, Yang Shengying, Luo Yong

**Affiliations:** ^1^West China Longquan Hospital Sichuan University, The First People’s Hospital of Longquanyi Chengdou District, Chengdu, China; ^2^Affiliated Hospital of Southwest Jiaotong University, The Third People's Hospital of Chengdu, Chengdu, China; ^3^The First People 's Hospital of Neijiang, Neijiang, China

**Keywords:** endometrial cancer, fertility preservation, hysteroscopy, tumor dissemination, case report

## Abstract

Endometrial cancer (EC) is a common gynecologic malignancy with a rising incidence in young women. While fertility-preserving progestin therapy is an option for early-stage, well-differentiated (Grade 1, FIGO IA) tumors, its efficacy in poorly differentiated (Grade 3) tumors remains controversial due to their aggressive nature and high recurrence rates. In this study, we report a 26-year-old nulliparous woman with Grade 3 endometrioid adenocarcinoma (FIGO IA) who underwent medroxyprogesterone acetate (MPA, 160 mg/day) therapy and three hysteroscopic biopsies within 10 months, each showing no residual malignancy. Shortly after the last hysteroscopy, she developed a rapidly enlarging adnexal mass, and imaging revealed extensive peritoneal metastases. Laparoscopic exploration confirmed widespread tumor dissemination, and despite paclitaxel plus cisplatin (TP regimen) chemotherapy, the disease progressed rapidly, demonstrating chemoresistance. She declined further treatment and succumbed to the disease within months. This case highlights the potential limitations and challenges of fertility-sparing treatment in high-grade endometrial carcinoma, even in FIGO IA stage, and underscores the importance of strict adherence to current selection criteria and thorough risk assessment. Additionally, it raises concerns about the potential role of repeated hysteroscopic procedures in tumor dissemination, particularly with high intrauterine pressure. Given the poor prognosis of Grade 3 endometrial carcinoma, early definitive surgery should be prioritized over conservative management. Further research is needed to evaluate the oncologic safety of repeated hysteroscopic procedures and explore alternative surveillance strategies.

## Introduction

Endometrial cancer (EC) is one of the most common gynecologic malignancies, with a rising incidence among young women, partly attributed to increasing obesity rates and lifestyle changes (1, 2). While the standard treatment for EC involves hysterectomy with or without lymphadenectomy, fertility-preserving approaches have gained attention for select patients with early-stage, low-grade tumors. Progestin-based therapy has demonstrated favorable outcomes in well-differentiated (Grade 1) endometrioid adenocarcinoma confined to the endometrium (FIGO IA) (2–4). However, its efficacy in poorly differentiated (Grade 3) tumors remains highly controversial due to their aggressive biological behavior, lower hormone receptor expression, and high recurrence rates (5, 6). Although current guidelines recommend conservative treatment only for Grade 1 tumors, some patients with high-grade disease still pursue fertility preservation, despite the potential risks of treatment failure and disease progression (7–9).

Recent molecular advances, notably The Cancer Genome Atlas (TCGA) classification, have defined four key subtypes of endometrial carcinoma—POLE-ultramutated, mismatch repair-deficient (MMR-d), no specific molecular profile (NSMP), and p53-abnormal—each with distinct prognostic and therapeutic implications (7). Among them, p53-abnormal tumors exhibit the worst prognosis and poor responsiveness to hormonal therapy, even in early-stage disease (7, 8). While fertility preservation is well established in early-stage, low-grade EC, its role in high-grade tumors remains controversial and insufficiently supported by evidence. The rising incidence of EC in reproductive-aged women highlights the growing dilemma of balancing fertility desires with biologically aggressive disease. This challenge is particularly acute in patients with FIGO IA, high-grade histology, and molecular high-risk features, where fertility-sparing treatment may appear feasible based on stage alone. However, it may be oncologically inappropriate due to tumor biology.

Hysteroscopic biopsy is widely used for the diagnosis and surveillance of EC, allowing direct visualization and targeted tissue sampling (10). However, concerns have been raised regarding its potential role in tumor dissemination, particularly in poorly differentiated carcinomas. Several studies suggest that the use of distension media and intrauterine pressure during hysteroscopy may facilitate retrograde tumor cell spread through the fallopian tubes, potentially leading to peritoneal dissemination (11, 12). While definitive evidence remains inconclusive, some reports indicate that high intrauterine pressure may increase the risk of exfoliated malignant cells being transported into the abdominal cavity, particularly in high-grade tumors with a greater propensity for peritoneal spread (13, 14). The safety of repeated hysteroscopic procedures in such cases remains uncertain, necessitating further investigation.

In this study, we report a rare case of a 26-year-old nulliparous woman diagnosed with poorly differentiated endometrioid adenocarcinoma (Grade 3, FIGO IA), who underwent repeated hysteroscopic biopsies while receiving progestin therapy as a fertility-preserving approach. Despite an initial histologic response, she subsequently developed rapid disease progression with adnexal and peritoneal metastases within a short timeframe. This case highlights the potential risks associated with fertility-sparing treatment in high-grade EC, particularly when guideline-based selection criteria are not strictly followed. Additionally, it raises concerns regarding the possible role of repeated hysteroscopic procedures in tumor dissemination, emphasizing the need for caution in the management of high-grade EC and further research into safer diagnostic and surveillance strategies.

## Case presentation

**Figure 1:**
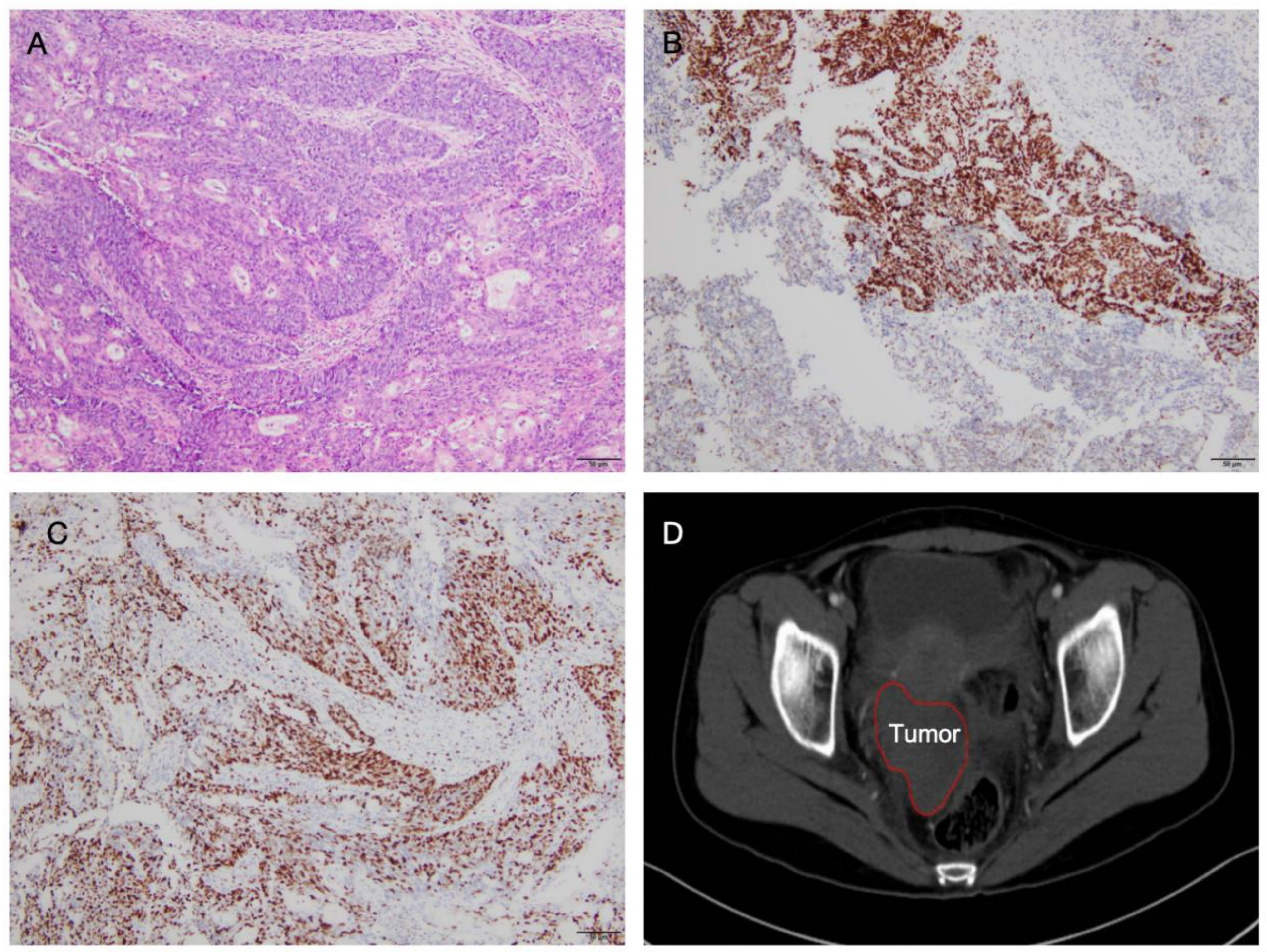
**(A)** Hematoxylin and eosin staining of a biopsy specimen showing poorly differentiated endometrioid adenocarcinoma (10× magnification). **(B)** Immunohistochemical staining demonstrating positive expression of P53 (10×). **(C)** Immunohistochemical staining showing a high Ki-67 proliferation index of approximately 80% (10×). **(D)** CT image revealing a solid pelvic mass measuring 7.1 × 5.7 × 4.6 cm.

A 26-year-old nulliparous woman presented with a 12-month history of irregular menstruation. In October 2023, the patient underwent a hysteroscopic biopsy for a suspected endometrial polyp. The procedure was performed using 5% glucose solution as the distension medium, with an intrauterine pressure of 80 mmHg. Histopathological examination unexpectedly revealed a poorly differentiated endometrioid adenocarcinoma (Grade 3) (Figure 1A). Immunohistochemical analysis showed positive for P53 (Figure 1B), a Ki-67 proliferation index of 80% (Figure 1C), and positive for Progesterone Receptor (PR), Estrogen Receptor (ER), and MutS Homolog 2 (MSH2). Following the pathological diagnosis, pelvic contrast-enhanced MRI was performed, revealing no evidence of myometrial invasion or cervical involvement. No enlarged pelvic or para-aortic lymph nodes or adnexal masses were detected. Based on these findings, the tumor was staged as FIGO IA. Total hysterectomy with or without lymphadenectomy was recommended as standard treatment per clinical guidelines. However, the patient strongly wished to preserve fertility. She received formal multidisciplinary counseling, during which the risks of fertility-sparing management—particularly in high-grade disease—were thoroughly discussed, including treatment failure, progression, and poor prognosis. Although molecular testing was offered, she declined further evaluation and opted for conservative treatment based on the existing pathological and immunohistochemical findings. After providing informed consent, she began oral medroxyprogesterone acetate (MPA) at 160 mg/day.

Three months after initiating MPA therapy, in January 2024, a follow-up hysteroscopic biopsy was performed under identical conditions. Histopathological examination revealed no residual malignancy, suggesting an initial response to hormonal therapy. Concurrent pelvic ultrasound showed no adnexal masses or abnormalities, and tumor markers were within normal ranges, with CA125 at 18.80 U/mL, CA199 at 33.47 U/mL (mildly elevated), and CEA at 0.75 ng/mL. Given the absence of detectable disease, MPA therapy was continued.

At the 6-month follow-up in June 2024, a repeat hysteroscopic biopsy again showed no evidence of malignancy, and pelvic ultrasound remained unremarkable. Tumor markers were stable, with CA125 at 18.80 U/mL, CA199 at 33.47 U/mL, and CEA at 0.75 ng/mL. However, just 3 weeks later, in July 2024, routine follow-up ultrasound unexpectedly detected a 4-cm right adnexal mass, despite the absence of abnormalities in prior imaging. The patient subsequently developed persistent lower abdominal pain, predominantly in the right lower quadrant. A repeat computed tomography (CT) on 20 July 2024 revealed a rapidly enlarging 7.1 × 5.7 × 4.6-cm solid pelvic mass with pelvic effusion, raising suspicion for tumor recurrence or metastasis (Figure 1D). At this time, CA125 was elevated to 36.80 U/mL, while CA199 (28.95 U/mL) and CEA (0.01 ng/mL) remained within normal limits.

Given the concerning findings, a PET/CT scan was performed, revealing an irregular hypermetabolic mass posterior to the uterus, with Fluorodeoxyglucose (FDG)-avid para-aortic and pelvic lymph nodes, findings consistent with metastatic disease. In August 2024, the patient underwent laparoscopic exploration, which was converted to laparotomy due to extensive peritoneal metastases. Intraoperative findings included multiple peritoneal nodules, right fallopian tube involvement, and invasion of the sigmoid colon serosa. A right salpingectomy, partial omentectomy, and pelvic mass resection were performed.

Postoperative pathology confirmed poorly differentiated endometrioid adenocarcinoma with extensive peritoneal involvement, with immunohistochemical staining showing P53 positivity, a Ki-67 proliferation index of 60%, PR positivity (30%), ER positivity (30%), and VIM positivity, findings consistent with the original uterine tumor.

In September 2024, the patient was started on paclitaxel plus cisplatin (TP regimen) chemotherapy. However, by October 2024, MRI demonstrated progressive disease, with an enlarging pelvic mass and multiple peritoneal nodules. Despite clear radiological evidence of disease progression, tumor markers remained within normal limits, with CA125 at 12.50 U/mL, CA199 at 23.68 U/mL, and CEA at 1.30 ng/mL. By November 2024, MRI showed tumor infiltration into the bowel and extensive pelvic adhesions, further suggesting chemoresistant disease. The patient was advised to seek surgical intervention at a higher-level center, but she declined further treatment. She was discharged with palliative care recommendations and succumbed to disease complications a few months later.

## Discussion

The histopathological and immunohistochemical comparison between the initial uterine tumor and the recurrent pelvic malignancy suggests that the recurrent disease was not a new primary tumor but rather a progression of the original carcinoma. Both tumors shared similar immunohistochemical markers, including P53 positivity and a high Ki-67 proliferation index, indicating that the tumor persisted despite hormonal therapy and later manifested as peritoneal metastases. This highlights the limitations of hormonal therapy in poorly differentiated tumors and raises concerns about the adequacy of fertility-preserving treatment in high-grade endometrial carcinoma.

According to clinical guidelines, fertility-preserving treatment is strictly recommended for patients with Grade 1 endometrioid adenocarcinoma confined to the endometrium (FIGO stage IA) and without lymphovascular invasion (7–9). In this case, the patient was diagnosed with a Grade 3 tumor, which falls outside the recommended criteria. However, due to her strong desire to retain reproductive potential, she declined comprehensive surgical staging and opted for conservative hormonal therapy. This decision likely contributed to the rapid progression of the disease. Although molecular classification was not performed, immunohistochemical staining revealed aberrant p53 expression and a Ki-67 index exceeding 80%, strongly suggesting a p53-abnormal molecular subtype. According to the recent systematic review, patients with p53-abn tumors demonstrated both lower complete response rates (50%) and higher recurrence rates (33.3%) following fertility-sparing treatment compared to the NSMP group (15). These findings support that the patient, despite having FIGO stage IA disease, was at high biological risk and therefore not an appropriate candidate for conservative management.

Furthermore, the study by Ferrari et al. underscores that molecular classification—particularly identification of p53-abn and MMRd/MSI-H subtypes—has prognostic value in guiding fertility-sparing decisions (15). While traditional criteria focus on grade and stage, molecular profiling provides an additional layer of risk stratification that can inform treatment selection and counseling. This case underscores the critical importance of adhering to established selection criteria and incorporating surrogate molecular markers such as p53 status and Ki-67 index into clinical decision-making. Clinicians should exercise caution and ensure thorough molecular and histopathological assessment before recommending fertility preservation in patients with high-grade or molecularly high-risk tumors.

Another critical consideration in this case is the potential role of repeated hysteroscopic procedures in tumor dissemination. The patient underwent three hysteroscopic biopsies within 10 months, raising concerns that retrograde tumor cell spread through the fallopian tubes into the peritoneal cavity may have contributed to the development of pelvic metastases. Although a direct causal relationship cannot be established based on a single case, this observation raises a biologically plausible concern, particularly in high-grade tumors with increased propensity for peritoneal dissemination.

Some studies have suggested that fluid irrigation and elevated intrauterine pressure during hysteroscopy may facilitate the trans-tubal transport of exfoliated malignant cells into the peritoneal cavity, especially in poorly differentiated tumors (13, 14). In this patient, serial ultrasonography during the hysteroscopic procedures showed no adnexal abnormalities, yet rapidly progressive adnexal and peritoneal metastases emerged within weeks following the third hysteroscopy. The procedures were performed using 5% glucose as the distension medium and an intrauterine pressure of 80 mmHg—within standard safety thresholds—but potentially sufficient to allow retrograde cell movement in the presence of patent tubes.

Nevertheless, existing literature remains inconclusive regarding the long-term clinical impact of hysteroscopy on tumor spread. While some reports have shown an increase in positive peritoneal cytology following hysteroscopy, multiple studies suggest that this does not translate into worse oncologic outcomes (16). This has led to the removal of peritoneal cytology from the 2023 FIGO staging criteria. However, in select high-grade or biologically aggressive cases, the theoretical risk warrants caution. Individualized risk–benefit assessment is essential when considering repeated hysteroscopic interventions for diagnostic or surveillance purposes in this population. Further prospective studies are needed to clarify whether hysteroscopy-related peritoneal dissemination has clinical relevance, particularly in the context of high-risk molecular subtypes.

The failure of chemotherapy in this case further underscores the need for novel therapeutic approaches. Standard chemotherapy regimens, such as paclitaxel plus cisplatin, may not be effective in all patients, particularly those with chemotherapy-resistant tumors. Molecular profiling of the tumor could have provided insights into potential alternative treatments, such as immune checkpoint inhibitors (pembrolizumab for MSI-H/dMMR tumors) or targeted therapies (such as PI3K/AKT/mTOR inhibitors in PTEN-mutated tumors) (17, 18). Given the poor prognosis associated with high-grade, hormone-resistant endometrial carcinoma, earlier consideration of molecular-targeted therapies might improve outcomes in similar cases.

This case highlights the importance of balancing fertility preservation with oncologic safety. Although conservative management is an attractive option for young patients, the risks of delayed radical intervention must be carefully evaluated, particularly in high-grade cases. The significantly worse prognosis of FIGO IA Grade 3 tumors compared to Grade 1 or 2 tumors, despite being in the same stage, demonstrates the crucial role of histologic grading in treatment decision-making. The higher recurrence rate and lower 5-year survival probability emphasize the need for strict patient selection criteria when considering fertility-preserving approaches (19). Future investigations should focus on identifying biomarkers predictive of hormonal therapy response, optimizing surveillance strategies for early recurrence detection, and exploring novel therapeutic interventions for aggressive endometrial carcinoma.

## Conclusion

In conclusion, this case illustrates the potential risks of fertility-preserving treatment in high-grade endometrial carcinoma, particularly when guideline-based selection criteria are not strictly followed. Even in FIGO IA stage, Grade 3 endometrial carcinoma carries a significantly worse prognosis than Grade 1/2 tumors, with a higher recurrence rate and lower long-term survival. Given these risks, early definitive surgery should remain the standard of care for high-grade tumors, and alternative molecular-based therapeutic strategies should be explored to improve outcomes in young patients with aggressive disease.

## Data Availability

The original contributions presented in the study are included in the article/supplementary material, further inquiries can be directed to the corresponding author.
